# Two new deraeocorine plant bug species from Japan (Heteroptera, Miridae, Deraeocorinae)

**DOI:** 10.3897/zookeys.796.21243

**Published:** 2018-11-15

**Authors:** Yukinobu Nakatani, Tomohide Yasunaga

**Affiliations:** 1 Division of Informatics and Inventory, Institute of Agro-Environmental Sciences, NARO, Kannondai 3-1-3, Tsukuba, Ibaraki 305-8604, Japan Institute of Agro-Environmental Sciences, NARO Tsukuba Japan; 2 Research Associate, Division of Invertebrate Zoology, American Museum of Natural History, New York, USA American Museum of Natural History New York United States of America

**Keywords:** Deraeocorinae, *
Fingulus
*, Heteroptera, Insecta, Japan, Miridae, new species, *
Stethoconus
*

## Abstract

Two new deraeocorine plant bug species, *Fingulushenrytomi* and *Stethoconustakaii*, are described from Japan. A color habitus image of live individuals and scanning electron micrographs are shown for each taxon to aid an unequivocal identification. A checklist and keys to species are also provided for Japanese *Fingulus* and *Stethoconus*.

## Introduction

The plant bug subfamily Deraeocorinae is the fifth largest in the family Miridae (Henry 2017). This group is defined by a toothed tarsal claw, hair-like parempodium (Figs 22, 27–28) and membranous endosoma, sometimes with sclerites on the lobes. The subfamily contains numerous predatory species that sometimes resemble their insect prey. Previous studies of the Japanese fauna of this subfamily include those by Miyamoto (1965) and Nakatani (1995, 1996), which have resulted in thirty-nine species being confirmed. The present paper documents two new species of the Deraeocorinae, *Fingulushenrytomi* (tribe Deraeocorini) and *Stethoconustakaii* (Hyaliodini), which were found in the temperate climate zones of southeastern Japan. Although the majority of the members of *Fingulus* Distant and *Stethoconus* Flor are thermophilic and known predominantly from the tropics and subtropics (Nakatani et al. 2000, Nakatani and Yasunaga 2001, Schuh 1995, 2002–2014, Stonedahl and Cassis 1991, Yasunaga et al. 1997, 2016), the new species extend the range of these genera farther north than expected. The new species are also considered predacious, as species belonging to *Fingulus* and *Stethoconus* in other regions are well known as predators (e.g., Henry et al. 1986; Neal et al. 1991; Wheeler 2001; Yasunaga et al. 1997, 2016). Color habitus images of live individuals and scanning electron micrographs are presented for the two new species and their closely related congeners.

## Materials and methods

Specimens used in this present work were deposited in American Museum of Natural History, New York, USA (**AMNH**); Institute of Agro-Environmental Sciences, NARO (**NIAES**) and T. Yasunaga Collection (**TYCN**). Terminal segments of the male abdomen were boiled in 5% KOH solution for 5 min to observe the genital structures. Matrix code labels are attached to the holotype and some representative specimens, which uniquely identify each specimen, and are referred to as ‘unique specimen identifiers’ (USIs). The USI codes [e.g., AMNH_PBI 012345] comprise an institution and project code (AMNH_PBI) and a unique number (012345). These data were digitized on the Arthropod Easy Capture (formerly the Planetary Biodiversity Inventory) database maintained by the American Museum of Natural History, New York, USA (http://research.amnh.org/pbi/) and are also searchable on ‘Heteroptera Species Pages’ (http://research.amnh.org/pbi/heteropteraspeciespage/). All measurements were made with an ocular micrometer and are given in millimeters. The synonymic lists for known taxa were omitted, as comprehensive catalogs are now available (Schuh 1995, 2002–2014; Kerzhner and Josifov 1999; Aukema et al. 2013 online catalog). Scanning electron micrographs were taken with a Hitachi Tabletop Microscope TM3030.

### Checklist of *Fingulus* and *Stethoconus* in Japan

**Genus *Fingulus* Distant, 1904**

*F.collaris* Miyamoto, 1965; Japan (Ryukyus; Ishigaki and Iriomote Islands), Laos, Thailand, India.

*F.henrytomi* sp. n.; Japan (Shikoku, Tsushima Island)

*F.longicornis* Miyamoto, 1965; Japan (Honshu, Shikoku, Kyushu, Ryukyus), Philippines (Mindanao)

*F.takahashii* Nakatani, Yasunaga & Takai, 2000; Japan (Ryukyus).

**Genus *Stethoconus* Flor, 1861**

*S.japonicus*, Schumacher, 1917: Japan (Honshu, Shikoku, Kyushu, Ryukyus), Russia (Primorye), Korea, China, USA (Maryland, adventive)

*S.praefectus* Distant, 1909: Japan (Ryukyus: Ishigaki and Iriomote Islands), China, Taiwan, India, Sri Lanka, USA (Florida, adventive)

*S.takaii* sp. n.: Japan (Honshu, Shikoku)

### Key to the Japanese species of *Fingulus* Distant

**Table:** 

1	Head paler than remainder of body; pronotal collar impunctate (Fig. 19); corium with pale markings	**2**
–	Dorsum uniform in coloration; pronotal collar punctate (Fig. 24)	**3**
2	Frons widely pale; yellowish-brown markings laterally at base of corium	*** F. henrytomi ***
–	Frons gradually paler anteriorly; apical part of corium narrowly pale	*** F. collaris ***
3	Dorsum blackish brown; femora and basal tibiae dark brown	*** F. longicornis ***
–	Dorsum reddish brown; legs entirely pale yellow	*** F. takahashii ***

### Key to the Japanese species of *Stethoconus* Flor

**Table:** 

1	Scutellum distinctly projecting, the tip extended posteriorly (Fig. 31)	*** S. praefectus ***
–	Scutellum somewhat weakly projecting, the tip not extended posteriorly	**2**
2	Antennal segment II yellowish brown with apical 1/3 darkened; a pair of yellowish-brown markings laterally on scutellum; mesepimeron mostly whitish yellow	*** S. japonicus ***
–	Antennal segment II entirely or basal and apical 1/3 dark; scutellum and mesepimeron entirely dark	*** S. takaii ***

### Results

#### Fingulus henrytomi sp. n.

Taxon classificationAnimaliaHeteropteraMiridae

http://zoobank.org/A0CE92C6-82DF-4C82-AB18-2975CEA5E64A

Figs 4 , 8–9 , 19–22

##### Type material.

Holotype: ♀, **Japan**: Shikoku, Kochi, Monobe, Nishikuma-keikoku, 5.VIII.2000, M. Takai (AMNH_PBI 00380591) (NIAES). **Paratype**: 1♀, Nagasaki, Tsushima Island, Mt. Tatera, 34°09'00"N, 129°13'30"E, 25 Sep 1993, T. Yasunaga (AMNH_PBI 00380592) (TYCN)

##### Diagnosis.

Dorsum dark brown with a pair of pale markings on hemelytra; head slightly pale; pronotum trapeziform, strongly convex; femora and bases of tibiae infuscate. In general appearance, this new species resembles *F.collaris* Miyamoto, from which it can be distinguished by its hemelytral coloration.

##### Description.

**Female**: Body dark chestnut brown with a pair of paler markings on hemelytra, highly polished and punctate. Head slightly paler than remainder of body, suddenly restricted anterior to compound eye; tip of tylus infuscate; jugum, lorum, and gena paler than frons; vertex somewhat depressed; postocular part elongate, neck-like; buccula tinged with red. Antennal segment I dark chestnut brown, apparently thicker than other segments; segments II–IV pale yellowish brown. Labium castaneous; apical 1/3 of segment III pale brown. Pronotum entirely dark chestnut brown, highly polished and punctate, trapeziform and strongly convex; posterior margin rounded; collar flattened and weakly punctate; prosternum somewhat pale, conically projecting; ostiolar peritreme whitish yellow. Hemelytra widely dark chestnut brown except marking on basal 1/3 of corium brown. Membrane infuscate adjacent to red-tinged vein. Femora dark reddish brown; tibiae pale yellow except base infuscate; tarsi pale yellow. Abdomen dark chestnut brown.

##### Measurements.

(♀). Total body length: 3.64–4.17; width head across eyes: 0.50–0.59; width vertex: 0.15; length labium: 1.23–1.38; length of antennal segments I–IV: 0.41–0.43, 1.07–1.11, 0.83–0.86, 0.49–0.50; length pronotum including collar: 1.10–1.18; width base of pronotum: 1.41–1.48; maximum width across hemelytra: 1.65–1.70; length of hind femur, tibia, and tarsus: 1.20–1.48, 1.47–1.55, 0.30–0.32.

##### Etymology.

Named in honor of Dr. Thomas J. (Tom) Henry, our honorable friend and mentor.

##### Distribution.

Japan (Shikoku: Kochi Pref., Tsushima Island).

##### Biology.

The habits of this new species remain unknown; the only information is that the type specimens were collected by using a UV light trap or sweeping broadleaf trees.

##### Remarks.

This new species can be distinguished from its congeners by the coloration described above. Based on the generally ovoid body and rather weakly porrect head, our new species is assumed to be most closely related to *F.collaris*. As in certain other congeners, the population density of *F.henrytomi* is extremely low, as only two females have been collected, in spite of our continuing efforts and those of our enthusiastic colleagues to find additional specimens.

**Figures 1–7.**
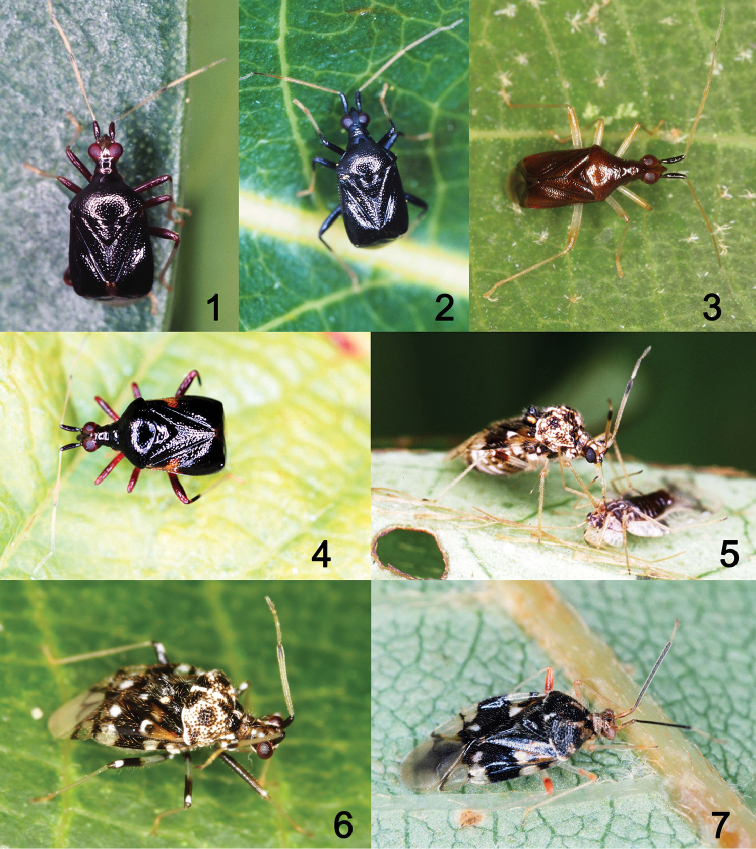
Habitus images of *Fingulus* spp. and *Stethoconus* spp. from Japan. **1***F.collaris* from Ishigaki Island. Ryukyus **2***F.longicornis* from Amami Island, Ryukyus **3***F.takahashii* from Ishigaki Island **4***F.henrytomi* sp. n. from Kochi, Shikoku, holotype, female **5***S.japonicus* preying on *Stephanitispyrioides* (Scott) (Tingidae), from Ibaraki, Honshu **6***S.praefectus* form Ishigaki Island **7***S.takaii* from Kochi, Holotype, Male.

#### Stethoconus takaii sp. n.

Taxon classificationAnimaliaHeteropteraMiridae

http://zoobank.org/10AB9347-913A-4842-9BF0-2777816555E2

Figs 7 , 10–11 , 13–14 , 16 , 18 , 25–29 , 32–37

Stethoconus japonicus : Yasunaga et al. 1997 (part), Nakatani and Yasunaga 2001(part).

##### Type material.

**Holotype**: ♂. **Japan**: Shikoku, Kochi., Agawa, Ino, 33.56N, 133.39E, 2 Aug 1998, M. Takai (AMNH_PBI 00380593) (NIAES). **Paratypes**: [Honshu] 1♀, Shizuoka, Atami alt. 300m, 22 Jul 1996, T. Ueda (NIAES); 1♂, Gifu, Gujou, Yamatocho-uchigatani, 28–29 Sep 2004, T. Ueda (NIAES); 1♂, Mie, Mt. Hirakura, 23 Jun 1953, Y. Miwa (NIAES); 2♂, Osaka, Minoo, 21 Jul 1995, K. Temma (NIAES); 1♂, Hyogo, Inagawa, Tsukunami, at light, 12 Jun 1997, Y. Nakatani (NIAES); 1♂, Nara, Kawakami, Shionoha, at light, 15 Jun 1993, Y. Nakatani (NIAES); 6♂1♀, Nara, Kawakami, Kitamata, at light, 15 Jun 1993, Y. Nakatani (AMNH & NIAES); 2♂, Nara, Kamikitayama, Mt. Wasamata, at light, 9 Aug 1995, T. Hirowatari & Y. Sawada (NIAES); 1♂: Wakayama, Kumanogawa, Doro Valley, Tamakiguchi, 33°53'53"N, 135°52'23"E, 15 Jun 1993, S. Gotoh (AMNH_PBI 00380594) (TYCN); 1♂, Wakayama, Shingu, Shirami, 9 Sep 1999, K. Temma (NIAES). [Shikoku] 1♀, same data as for holotype (TYCN).

**Figures 8–11.**
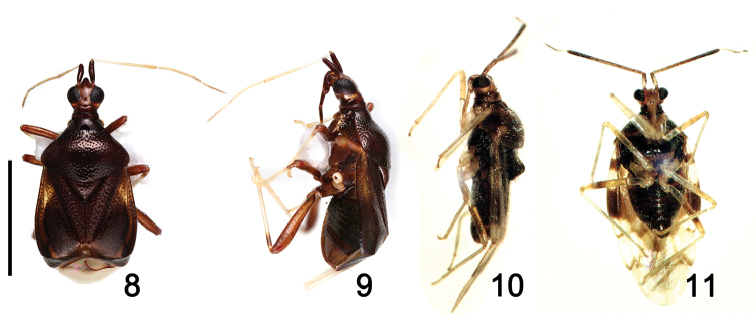
Holotype material of *F.henrytomi* (**8–9**) and *S.takaii* (**10–11**). Scale bar: 2mm.

##### Diagnosis.

Coloration generally dark; antennal segment II, or at least basal and apical 1/3, darkened; areas between pronotal punctures rather smooth; scutellum weakly elevated and blackish; mesepimeron blackish; abdomen mostly blackish.

##### Description.

Body generally maculate; dorsum shiny, densely clothed with long erect yellowish setae. Head shiny yellowish brown; brown stripe on vertex. Antenna dark brown; basal 1/3 or half of segment I somewhat pale, sometimes tinged with red; middle portion of segment II sometimes pale; base of segment III pale. Labium yellowish brown except tip brown, reaching anterior margin of mesosternum. Pronotum dark brown with yellowish quotation mark-shaped markings, strongly convex and distinctly punctate, densely covered with long erect yellowish setae; collar yellowish brown with dark base punctate, length 0.56 of width; triangular yellowish marking on mesal calli; short longitudinal whitish stripe on base of disc; posterior margin narrowly pale; areas between punctures on disc somewhat swollen but not calloused; prosternum yellowish brown, conically projecting. Scutellum entirely dark, gradually elevated to posterior with rounded carinate process; meso- and metapleura dark except for ostiolar peritreme whitish yellow. Hemelytron smooth, shiny and transparent with two transverse brown bands; anterior 1/4 and posterior half dark brown; mesal half of posterior end of corium brown, both sides of marking connected with posterior transverse band; posterior part of embolium narrowly brown; posterior part of cuneus and membrane veins tinged with brown. Legs pale yellowish brown; hind femur with apical 1/3 brown or with red band. Abdomen almost entirely dark brown in male, lateral half of segments II, IV and VII yellowish brown in female; marking on female segment II convex. Male genitalia as in Figs 32–37. Sensory lobe of left paramere slightly swollen near base; right paramere simple; endosoma with spiculate sclerite at base, lacking lobal sclerite.

**Figures 12–18.**
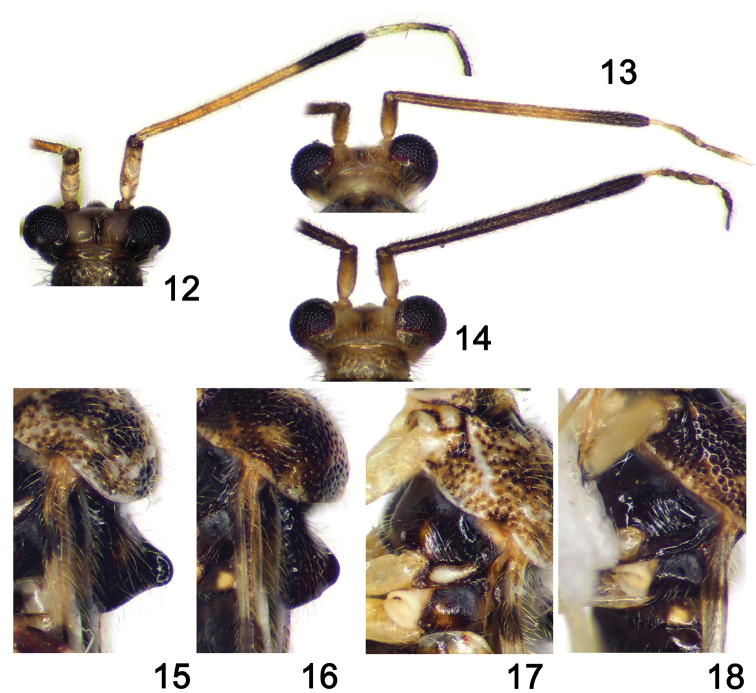
Magnified images of *Stethoconus* species. **12–14** Heads and antennae **15–16** scutellum, lateral aspects **17–18** pleura **12, 15, 17***S.japonicus***13–14, 16, 18***S.takaii*.

##### Measurements

(♂/♀). Total body length: 3.70–4.45/4.19–4.62; width head across eyes: 0.74–0.80/0.75–0.85; width vertex: 0.28–0.30/0.33–0.37; length of labium; 0.92–0.97/0.98–1.10; length of antennal segments I–IV: 0.33–0.38/0.44–0.50, 1.40–1.58/1.44–1.65, 0.38–0.43/0.44–0.60, 0.33–0.41/0.33–0.36; length of pronotum including collar: 1.00–1.08/1.0–1.26; width base of pronotum: 1.45–1.60/1.66–1.81; maximum width across hemelytra: 1.80–20.4/2.04–2.21; length of hind femur, tibia, and tarsus: 1.28–1.45/1.52–1.68, 1.69–2.01/1.61–2.18, 0.27–0.30/0.29–0.36.

##### Distribution.

Japan (Shikoku: Kochi Pref., Tsushima Island).

##### Etymology.

Named after Mr. Mikio Takai, who first suggested the presence of this new species.

##### Biology.

According to Mr. M. Takai (pers. obs.), *Stethoconustakaii* was associated with a colony of *Stephanitisyasumatsui* Takeya, 1951 (Tingidae) on an evergreen broadleaf tree, *Trochodendronaralioides* Siebold et Zucc. (Trochodendraceae). This tingid species is assumed to be a prey item for the mirid.

**Figures 19–24.**
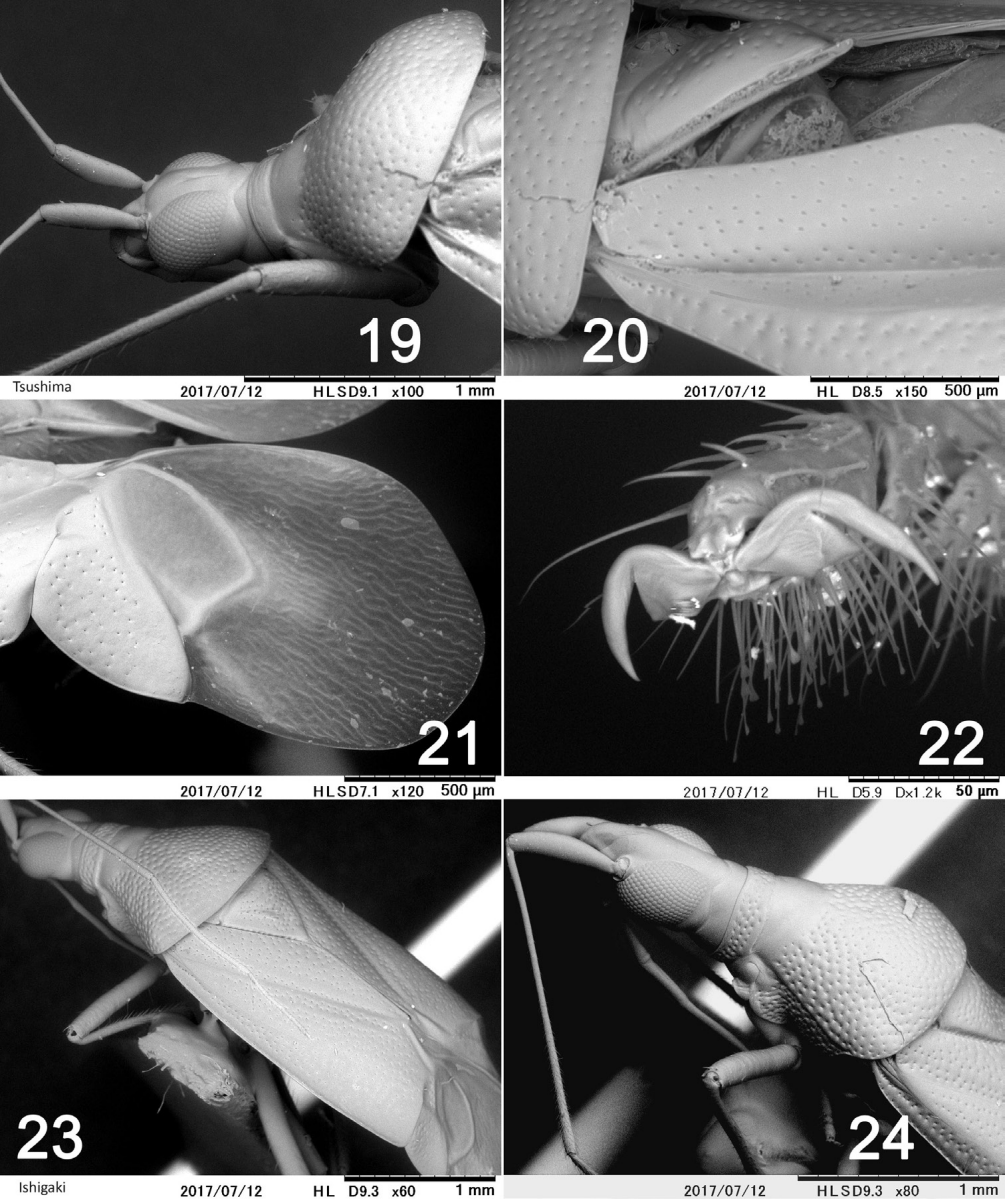
Scanning electron micrographs of *Fingulus* species. **19–22***F.henrytomi***23–24***F.takahashii***19, 24** head and pronotum **20, 23** hemelytra **21** membrane **22** tarsal claw.

##### Remarks.

**Figures 25–31.**
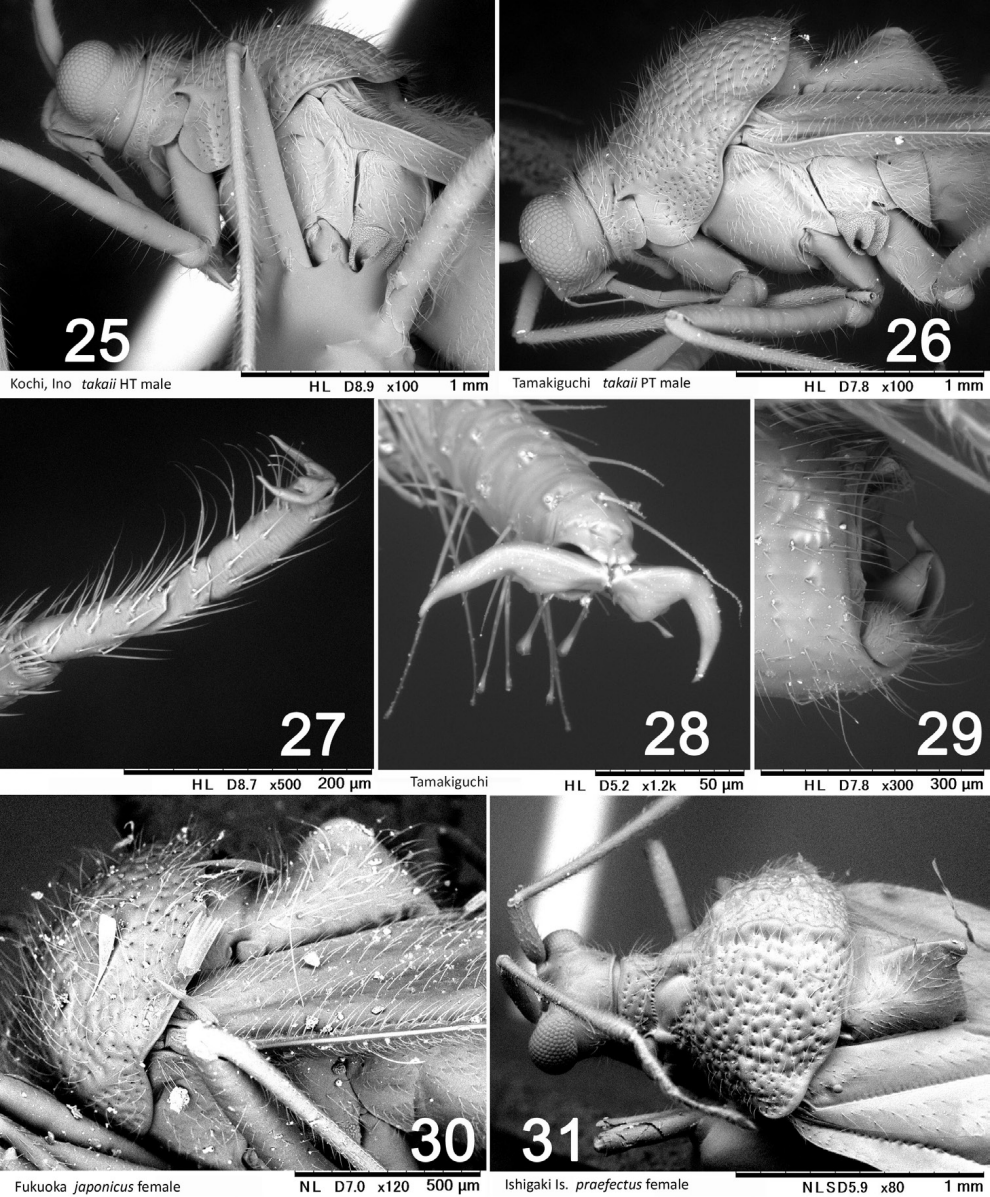
Scanning electron micrographs of *Stethoconus* species. **25–29***S.takaii***30***S.japonicus***31***S.praefectus***27** tarsus **28** tarsal claw **29** terminal abdomen.

The male genital structure of this new species is similar to that of *S.japonicus* except for the shape of the left paramere. *Stethoconustakaii* can be distinguished from the latter by the following characters. Antennal segment II dark, if middle pale, at least basal 1/3 infuscate (Figs 12–14); areas between punctures on pronotum weakly swollen, but not calloused (Figs 25–26, 30–31); scutellum weakly elevated and dark without marking (Figs 15–16); pale marking on mesepimeron absent (Figs 17–18); basal part of left paramere somewhat swollen. Some specimens of the new species have been misidentified as *S.japonicus* (Yasunaga et al. 1997, Nakatani and Yasunaga 2001). The description and illustration by Nawa (1910), on which the specific name *S.japonicus* was based (Schumacher 1917; see also Yasunaga et al. 1996), unequivocally correspond to what has been identified as *S.japonicus* (with only an apically infuscate antennal segment II and a yellow marking on scutellum).

**Figures 32–37.**
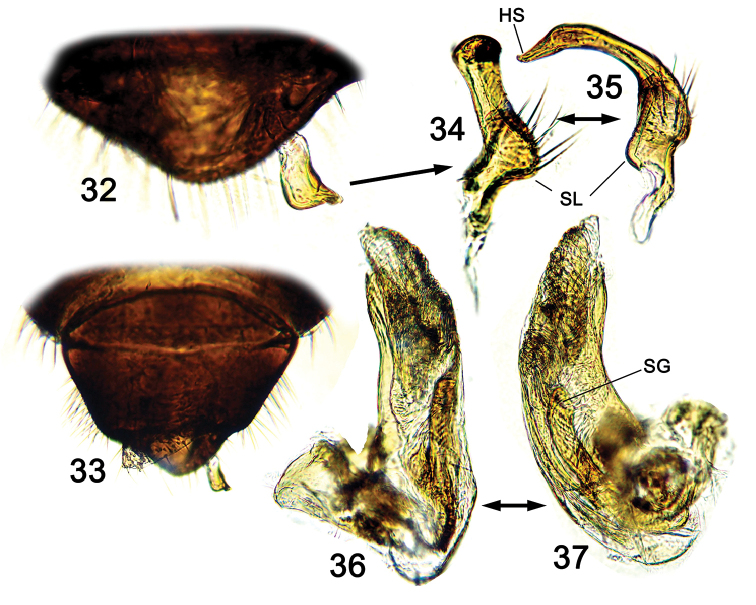
Male genitalia of *S.takaii*. **32–33** Pygophore **34–35** left paramere **36–37** endosoma. Abbreviations: HS = hypophysis, SL = sensory lobe, SG = Secondary gonophore.

## Supplementary Material

XML Treatment for Fingulus henrytomi

XML Treatment for Stethoconus takaii
